# Microstructure and Cryogenic Mechanical Properties of a Heterostructured Al_11_Cr_14_Fe_50_Ni_25_ High-Entropy Alloy Processed by Short-Time Annealing

**DOI:** 10.3390/ma19122582

**Published:** 2026-06-15

**Authors:** Zhe Song, Xixi Qi, Zhong Wang, Yiming Lai, Yuyang Chen, Yuefei Jia, Qi Yang, Xiaodong Wang

**Affiliations:** 1 AECC Chengdu Enging Co., Ltd., Chengdu 610080, China; 2 State Key Laboratory of Metal Matrix Composites, Shanghai Jiao Tong University, Shanghai 200240, China; 3 Shanghai Spaceflight Precision Machinery Institute, Shanghai 200200, China; 4 Guangdong Provincial Key Laboratory for Processing and Forming of Advanced Metallic Materials, South China University of Technology, Guangzhou 510640, China; 5 State Key Laboratory of Materials for Advanced Nuclear Energy, Shanghai University, Shanghai 200444, China; 6 Shanghai Key Laboratory of Engineering Materials Application and Evaluation, Shanghai Research Institute of Materials Co., Ltd., Shanghai 200200, China; 7 Shanghai Key Laboratory of Hydrogen Science, Center of Hydrogen Science, School of Materials Science and Engineering, Shanghai Jiao Tong University, Shanghai 200240, China

**Keywords:** high-entropy alloy, Co-free alloy, heterostructured microstructure, cryogenic properties, HDI strengthening

## Abstract

Developing low-cost, Co-free high-entropy alloys (HEAs) that retain both high strength and useful ductility at cryogenic temperatures remains challenging because hard strengthening phases usually intensify strain localization and accelerate plastic instability. In this work, a Fe-enriched Al_11_Cr_14_Fe_50_Ni_25_ HEA was designed and processed by heavy cold rolling followed by short-time annealing at 900 °C for 10 min to construct a hierarchical heterogeneous microstructure. The alloy consists of an FCC-dominated matrix and an ordered B2 phase distributed in recrystallized and unrecrystallized domains over multiple length scales. Tensile testing shows that the alloy achieves a yield strength of 953 MPa, an ultimate tensile strength of 1160 MPa, and an elongation of 21.1% at 298 K, while these values increase to 1268 MPa, 1686 MPa, and 28.6%, respectively, at 77 K. Load–unload–reload analysis at 77 K reveals that the hetero-deformation-induced stress reaches about 804 MPa at a true strain of 25%, contributing more than 52% of the total flow stress. The superior cryogenic strength–ductility synergy is attributed to strain partitioning between soft FCC and hard B2 phases and between recrystallized and unrecrystallized regions, which promotes geometrically necessary dislocation accumulation, back-stress strengthening, and sustained work hardening. This study demonstrates that hierarchical heterostructure design provides an effective route for developing cost-conscious Co-free HEAs for cryogenic structural applications.

## 1. Introduction

Structural materials used in liquefied natural gas (LNG) storage and transportation [1,2], polar marine engineering [3,4], and cryogenic aerospace systems are commonly exposed to complex loading conditions, including tensile, impact, and cyclic stresses, under prolonged low-temperature service. Although decreasing temperature generally increases material strength, it often causes a pronounced loss of ductility, thereby introducing considerable safety concerns [5,6]. Consequently, achieving a desirable combination of high strength and high ductility under extremely low-temperature conditions remains a critical challenge in the design of cryogenic structural materials.

High-entropy alloys (HEAs) [7,8], also known as multi-principal-element alloys [9,10], have opened new possibilities for overcoming the compositional and microstructural limitations of conventional alloys [7,11]. Owing to their high compositional flexibility and tunable phase stability [12], HEAs provide unique opportunities to optimize strength and ductility synergistically through solid-solution strengthening [13,14], severe lattice distortion [15], and tailored phase constitutions [16,17]. In particular, several HEA systems exhibit high work-hardening capability and excellent fracture toughness at cryogenic temperatures [18,19], indicating strong potential for low-temperature structural applications [20,21]. Nevertheless, from the viewpoint of microstructural design, single-phase multi-principal-element alloys often suffer from an intrinsic trade-off between strength and ductility. Single-phase FCC alloys generally possess excellent ductility but relatively limited yield strength [22,23], whereas single-phase BCC alloys exhibit high strength but poor ductility, even at room temperature and especially under cryogenic conditions [24,25].

To overcome this strength–ductility trade-off, increasing attention has been directed toward dual-phase and multiphase HEAs. However, even when dual-phase strengthening, grain refinement, or precipitation strengthening is introduced, the limited dislocation mobility and strain localization that occur at low temperatures still hinder the achievement of an optimal strength–ductility balance. For example, Xu et al. [26] reported a dual-phase BCT + FCC alloy with a tensile strength of approximately 811 MPa at room temperature but with an elongation of only ~10%. Similarly, Peng et al. [27] increased the yield strength of a single-phase FCC alloy to ~1040 MPa through nanoscale M_23_C_6_ precipitation, whereas the elongation remained limited to ~12%. Such property combinations are insufficient for more demanding service environments. These examples indicate that conventional microstructural design strategies alone are inadequate for simultaneously achieving high strength and high ductility under cryogenic conditions.

To address this issue, recent studies have increasingly focused on microstructural architectures capable of sustaining strong work hardening, among which heterogeneous structures are particularly promising. Heterogeneous structures can generate hetero-deformation-induced strengthening and enhanced work hardening during plastic deformation, thereby enabling materials to retain useful ductility even at high strength levels. For instance, Du et al. [28] achieved a tensile strength of approximately 2.2 GPa together with an elongation of 13% at room temperature by introducing a heterogeneous structure consisting of a matrix and precipitates, substantially outperforming homogeneous counterparts. Wu and Zhang [29] also demonstrated that non-equiatomic multi-principal-element alloys, such as AlCoCrFeNi and (CoCrNi)_88_Al_10_Ta_2_, can exhibit superior strength–ductility combinations, with particularly pronounced improvements at low temperatures.

Despite these advances, many HEAs exhibiting excellent cryogenic strength–ductility synergy rely on expensive alloying elements such as Co and V, which inevitably restrict their economic viability and engineering applicability. Previous studies have shown that increasing the Fe content can destabilize the FCC phase and facilitate phase transformations, thereby promoting the formation of complex multiphase structures and introducing multiple strengthening mechanisms [30,31]. Accordingly, adjusting the Fe content offers an effective strategy for tailoring the mechanical properties of HEAs at both room and cryogenic temperatures. Furthermore, due to its natural abundance and low smelting cost, Fe is among the most economical metallic elements. Therefore, substituting Co with higher Fe content has emerged as a key design strategy—both theoretically and experimentally—for developing low-cost, high-performance HEAs [32].

Guided by this concept, a Co-free Al_11_Cr_14_Fe_50_Ni_25_ HEA was designed with an increased Fe content of 50 at% to explore the tensile behavior of low-cost HEAs at room and cryogenic temperatures. The Cr and Al contents were fixed at 14 at% and 11 at%, respectively, to ensure adequate corrosion and oxidation resistance. Furthermore, a multiscale heterogeneous microstructure was successfully established via heavy cold rolling followed by short-time annealing at 900 °C for 10 min. The phase constitution, microstructure, and defect structure were systematically characterized using synchrotron high-energy X-ray diffraction (HEXRD), scanning electron microscopy/energy-dispersive spectroscopy (SEM/EDS), transmission electron microscopy (TEM), and electron backscatter diffraction (EBSD). Furthermore, the microstructural evolution, tensile behavior, and underlying strengthening and toughening mechanisms were investigated through quasi-static tensile testing at 298 K and 77 K, combined with load–unload–reload (LUR) tensile tests.

## 2. Materials and Experimental Methods

**Figure 1:**
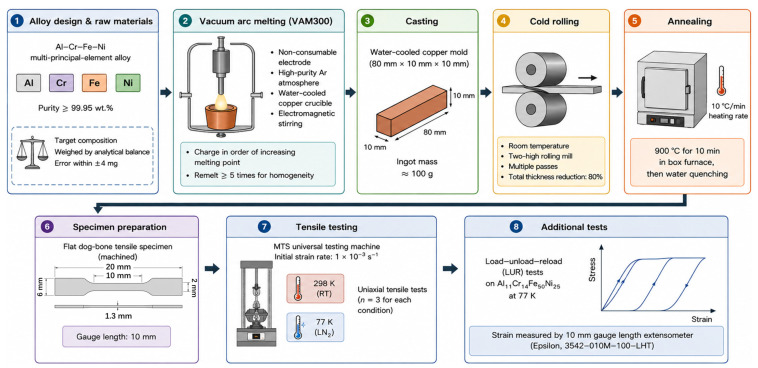
Detailed experimental workflow.

Al, Cr, Fe, and Ni were selected as constituent elements to prepare the target multi-principal-element alloy. The basic physical and chemical parameters of each element are listed in Table 1. The comparison of raw material cost between this alloy and the others is shown in Figure 1. Alloy ingots were fabricated using a non-consumable vacuum arc melting furnace (Micro-tec., VAM300, Shenyang, China). High-purity elemental Al, Cr, Fe, and Ni bulks with purities ≥ 99.95 wt.% were used as raw materials. The mass of each ingot was controlled at approximately 100 g. The mass of each element was calculated according to the target composition and weighed using a high-precision analytical balance, with the weighing error controlled within ±4 mg. The raw metals were placed in a water-cooled copper crucible in the order of increasing melting point. Melting was carried out under a high-purity argon atmosphere, and each ingot was remelted at least five times to ensure compositional homogeneity. Electromagnetic stirring was applied during melting to promote convection in the molten pool. After remelting, the alloy was cast into a water-cooled copper mold with dimensions of 80 mm × 10 mm × 10 mm.

Cold rolling was performed at room temperature using a two-high rolling mill. The samples were rolled along a fixed rolling direction through multiple passes, and the total thickness reduction reached 80%. The cold-rolled specimens were then annealed at 900 °C for 10 min in a box furnace, followed by water quenching to suppress further microstructural evolution during cooling. And the heating rate is 10 °C/min. The alloy composition obtained via Inductively Coupled Plasma (ICP) spectroscopy was determined to be approximately Al_11.6_Cr_14.4_Fe_49.7_Ni_24.3_ (at.%). Equilibrium phase diagram calculations were conducted using Thermo-Calc 2024a software with the TCHEA5.0 database.

Flat dog-bone tensile specimens with gauge dimensions of 10 mm × 2 mm × 1.3 mm were machined for tensile testing. Uniaxial tensile tests were carried out at room temperature (298 K) and liquid-nitrogen temperature (77 K) using an universal testing machine (MTS, CBT1504, Eden Prairie, MN, USA) at an initial strain rate of 1 × 10^−3^ s^−1^. To ensure reproducibility, each tensile test was repeated three times. Load–unload–reload (LUR) tests were performed on the Al_11_Cr_14_Fe_50_Ni_25_ alloy at 77 K. The strain during both monotonic tensile and LUR tests was measured using a 10 mm gauge length extensometer (Epsilon, 3542-010M-100-LHT, Irving, TX, USA). To provide a clearer description of the methodology and enhance reproducibility, the detailed experimental workflow is summarized in Figure 1.

The synchrotron high-energy X-ray diffraction (HEXRD) experiments in this study were conducted at the P21 beamline of the PETRA III high-energy synchrotron radiation source at Deutsches Elektronen-Synchrotron (DESY) (Altona, Germany). A flat-panel detector (Varex, XRD4343CT, Salt Lake City, UT, USA, 2880 × 2880 pixels) was employed to collect the diffraction data, and the detector parameters were calibrated using a CeO_2_ standard sample. The synchrotron X-ray beam had a spot size of 200 × 200 μm^2^, with a photon energy of 52 keV, corresponding to a wavelength of 0.2386 Å. The two-dimensional diffraction data were reduced to one-dimensional patterns and quantitatively analyzed using GSAS-II, developed at the Advanced Photon Source (APS), Lemont, IL, USA.

The microstructure was characterized using field-emission scanning electron microscopy (FE-SEM, ZEISS Sigma 560, ZEISS, Oberkochen, Germany) equipped with electron backscatter diffraction (EBSD, Oxford Symmetry S3) and energy-dispersive X-ray spectroscopy (EDS, Oxford Xplore 65, Buckinghamshire, UK) detectors. For SEM and EBSD analyses, the specimens were mechanically polished and subsequently electropolished at 25 V for 60 s in a solution of 92 vol.% CH_3_COOH and 8 vol.% HClO_4_. Detailed microstructural characterization was further conducted using transmission electron microscopy (TEM, Talos F200X, Thermo Fisher Scientific, Waltham, MA, USA). TEM specimens were ground using 600#–2000# SiC abrasive papers to a thickness of approximately 70 μm, followed by twin-jet electropolishing to achieve electron transparency. The electrolyte consisted of 10 vol.% HClO_4_ and 90 vol.% ethanol.

## 3. Results

### 3.1. Phase Compositions

**Figure 2:**
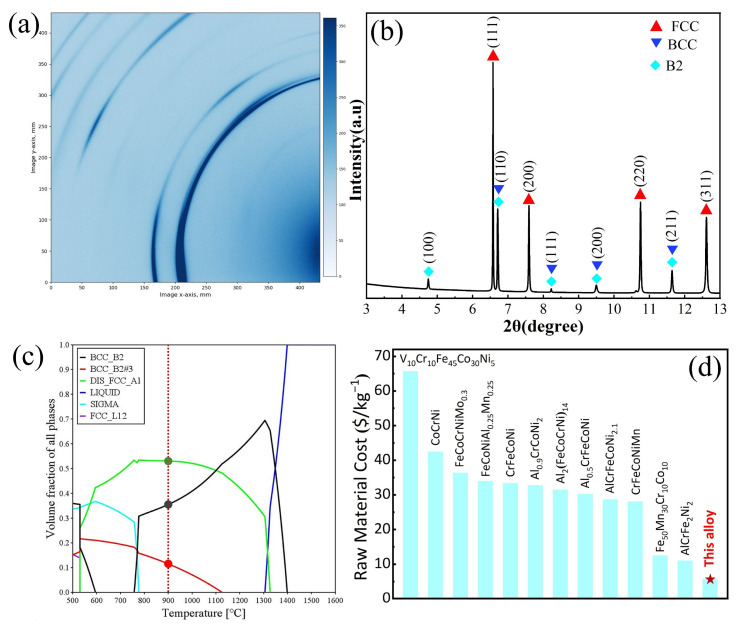
Synchrotron high-energy X-ray diffraction patterns of the Al_11_Cr_14_Fe_50_Ni_25_ HEA after short-time annealing at 900 °C: (**a**) raw 2D pattern; (**b**) integrated 1D profiles. (**c**) CALPHAD calculation results for the Al_11_Cr_14_Fe_50_Ni_25_ alloy. (**d**) Comparison of raw material costs between conventional HEAs and the present alloy.

The raw 2D diffraction patterns and the corresponding integrated 1D profiles of the synchrotron high-energy X-ray diffraction measurements for the Al_11_Cr_14_Fe_50_Ni_25_ HEA after short-time annealing at 900 °C are shown in Figure 2a,b. The HEXRD pattern of the Al_11_Cr_14_Fe_50_Ni_25_ alloy exhibits diffraction peaks primarily associated with the FCC and BCC phases. Notably, the diffraction intensity of the FCC peaks is significantly higher than that of the BCC peaks, indicating that the FCC phase is dominant, with a phase fraction of approximately 73.6%, while the BCC phase accounts for about 26.4%. Furthermore, a distinct superlattice reflection corresponding to the (100) plane is observed, which is characteristic of the ordered B2 phase. This observation confirms that the BCC phase in the alloy consists of both disordered BCC (A2) and ordered B2 phases.

Based on the measured FCC lattice parameter of 0.3552 nm, the calculated *d*-spacings for FCC (111), FCC (200), and FCC (220) are 0.2051, 0.1776, and 0.1256 nm, respectively. For the ordered B2 phase with a lattice parameter of about 0.2831 nm, the calculated d-spacings for B2 (100), B2 (110), and B2 (200) are 0.2831, 0.2002, and 0.1416 nm, respectively. The presence of the B2 (100) superlattice reflection confirms chemical ordering. The calculated atomic-size mismatch parameter is 4.34%, the electronegativity difference is 0.101, and the VEC is 7.67 for the nominal Al_11_Cr_14_Fe_50_Ni_25_ composition, indicating a strong tendency to form an FCC-dominated structure together with ordered B2 due to Al-Ni chemical affinity and lattice distortion.

As shown in Figure 2c, CALPHAD equilibrium calculations predict that the Al_11_Cr_14_Fe_50_Ni_25_ alloy lies within the three-phase region of FCC, ordered BCC (B2), and disordered BCC (A2) at 900 °C, with corresponding equilibrium volume fractions of 52.4%, 11.4%, and 36.2%, respectively. The FCC phase fraction determined by HEXRD measurements is substantially higher than that predicted by the equilibrium phase diagram. This discrepancy between the CALPHAD prediction and the HEXRD-derived phase fraction can be attributed to both kinetic effects and limitations of the thermodynamic database. The CALPHAD result represents the equilibrium state at 900 °C, whereas the experimentally observed microstructure was formed under non-equilibrium conditions, involving solidification, severe cold rolling, a short 10 min annealing treatment, and subsequent water quenching. The formation and coarsening of ordered B2/A2 phases require long-range substitutional diffusion of Al, Ni, Cr, and Fe. Such diffusion is strongly time-dependent and is unlikely to reach equilibrium within the present short annealing window. Water quenching further suppresses post-annealing diffusion and preserves an FCC-dominated metastable dual-phase structure. Therefore, the higher experimental FCC fraction does not contradict the thermodynamic driving force for multiphase equilibrium but rather reflects the kinetically arrested microstructure designed in this work. Additional annealing-time experiments and database cross-checks would further clarify the time-dependent approach toward equilibrium and will be considered in future work. Figure 2d compares the raw material costs of conventional HEAs with the Al_11_Cr_14_Fe_50_Ni_25_ HEA. Eliminating Co and increasing Fe content reduces the alloy cost by more than half compared to typical Co-containing HEAs. Moreover, the alloy can be produced via simple cold rolling followed by a 10 min anneal, enabling a highly efficient, low-energy fabrication process.

### 3.2. Microstructure

**Figure 3:**
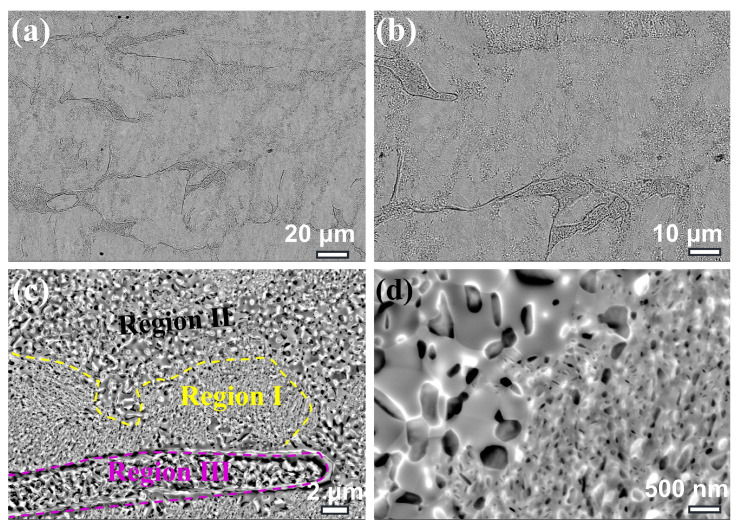
SEM images of the short-time annealed Al_11_Cr_14_Fe_50_Ni_25_ HEA. (**a**,**b**) low-magnification SEM images, (**c**,**d**) high-magnification SEM images.

The short-time annealed Al_11_Cr_14_Fe_50_Ni_25_ HEA exhibits a typical hierarchical heterogeneous microstructure. Figure 3 presents the SEM images of the alloy after short-time annealing. As shown in Figure 3a,b, regions with pronounced morphological heterogeneity are clearly observed, indicating the coexistence of recrystallized and unrecrystallized domains. The higher-magnification image in Figure 3c,d further reveals the coexistence of coarse- and fine-grained regions, confirming the formation of a heterogeneous microstructure over multiple length scales. Based on the morphological characteristics and phase constitution, the microstructure can be divided into three representative regions, denoted as Region I, Region II, and Region III, as marked in Figure 3c.

**Figure 4:**
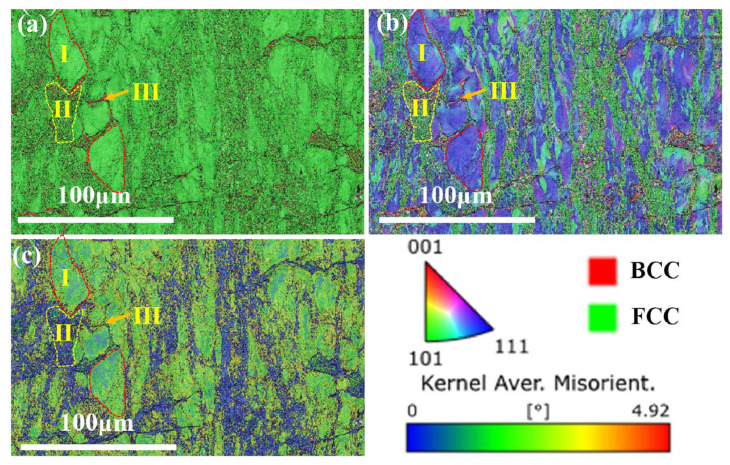
(**a**) EBSD inverse pole figure (IPF) map, (**b**) band contrast (BC) map, and (**c**) kernel average misorientation (KAM) map of the short-time annealed Al_11_Cr_14_Fe_50_Ni_25_ HEA.

The EBSD phase map (Figure 4a) shows that Regions I and II are both dominated by the FCC phase. Region I consists of elongated grains containing a high density of nanoscale precipitates and exhibits a certain degree of texture (Figure 4b), which is characteristic of an unrecrystallized microstructure. In contrast, Region II is mainly composed of fine equiaxed grains, indicative of a recrystallized microstructure. Region III exhibits a distinct core–shell morphology, with the BCC phase as the dominant constituent. The KAM map (Figure 4c) further reveals pronounced differences among the three regions in terms of local misorientation and dislocation accumulation. Region I exhibits high KAM values generally, indicating a high density of geometrically necessary dislocations (GNDs). This is consistent with its elongated grain morphology and unrecrystallized character, suggesting substantial retained strain in this region. By contrast, Region II shows significantly lower KAM values and thus a lower GND density, indicating that recrystallization has effectively eliminated most dislocations and related crystalline defects, resulting in a relatively low-strain state. Although Region III also exhibits high KAM values and a high GND density, no obvious deformation-induced grain elongation is observed. This suggests that the orientation gradients in this region are more likely associated with strain incompatibility with the surrounding FCC matrix, rather than being generated by plastic deformation within Region III itself.

**Figure 5:**
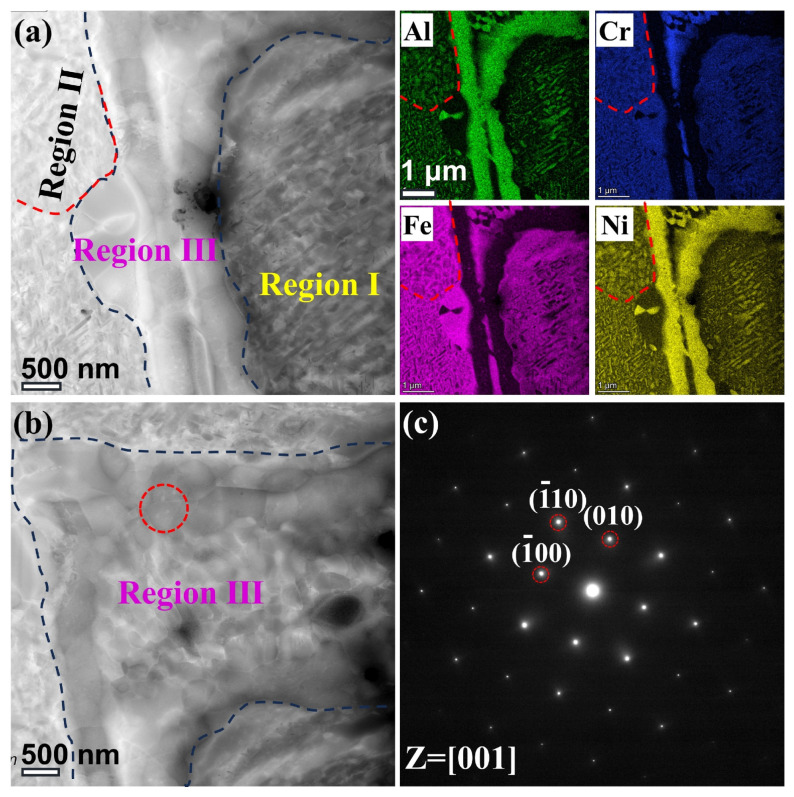
(**a**) BF-STEM image and corresponding EDS elemental maps of the short-time annealed Al_11_Cr_14_Fe_50_Ni_25_ HEA; (**b**) BF-TEM image of Region III; (**c**) SAED pattern of Region III along the [001] zone axis.

Figure 5a presents the BF-STEM images and corresponding EDS elemental maps of the three characteristic regions in the short-time annealed Al_11_Cr_14_Fe_50_Ni_25_ alloy, revealing clear differences in both chemistry and structural features. Region I corresponds to the unrecrystallized domain, which is characterized by a high dislocation density and a large number of rod-like precipitates. EDS analysis shows that these rod-like precipitates are enriched in Ni and Al, whereas the matrix is enriched in Cr and Fe. Both Regions II and III consist of recrystallized nano-equiaxed grains; however, they differ markedly in phase constitution and precipitate distribution. Region II is composed of two types of equiaxed grains with complementary compositions, i.e., an Al-Ni-rich precipitate phase and an Fe-Cr-rich matrix phase. Region III, by contrast, displays a core–shell structure, in which the shell is mainly composed of Al- and Ni-rich equiaxed grains, together with a small number of Fe- and Cr-rich nanoscale precipitates dispersed within the shell (Figure 5b). To identify the crystal structure of the shell region, selected-area electron diffraction (SAED) was further performed. As shown in Figure 5c, the diffraction pattern taken along the [001] zone axis exhibits distinct superlattice reflections, consistent with the ordered B2 structure, with a calculated lattice parameter of 0.2869 nm.

**Figure 6:**
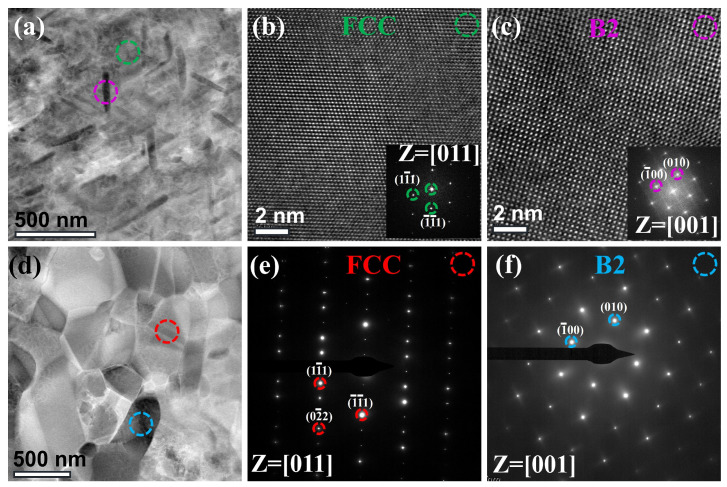
(**a**) BF-TEM image of the precipitates in unrecrystallized Region I; (**b**) HRTEM image and corresponding FFT pattern of the matrix in Region I along the [011] zone axis; (**c**) HRTEM image and corresponding FFT pattern of the precipitates in Region I; (**d**) BF-TEM image of recrystallized Region II; (**e**,**f**) SAED patterns of the selected areas in Region II.

To further clarify the structural nature of the rod-like precipitates in Region I, high-resolution TEM analyses were conducted (Figure 6). The SAED pattern of the rod-like precipitates in the unrecrystallized region along the [011] zone axis (Figure 6b), together with the HRTEM lattice image (Figure 6c), confirms that these precipitates are an ordered B2 phase, with a lattice parameter of approximately 0.2831 nm. Region II is composed of nano-equiaxed grains (Figure 6d), and SAED analysis (Figure 6f) demonstrates that this region consists of an FCC + ordered B2 dual-phase microstructure. Annealing twins are observed within the FCC phase. The lattice parameters of the FCC and ordered B2 phases are 0.3552 nm and 0.2831 nm, respectively, indicating a substantial lattice mismatch and hence a non-coherent interface between the two phases. Overall, both the unrecrystallized and recrystallized regions consist of FCC and ordered B2 phases; however, they differ significantly in morphology and length-scale distribution, thereby collectively giving rise to the hierarchical heterogeneous microstructure.

### 3.3. Tensile Properties

**Figure 7:**
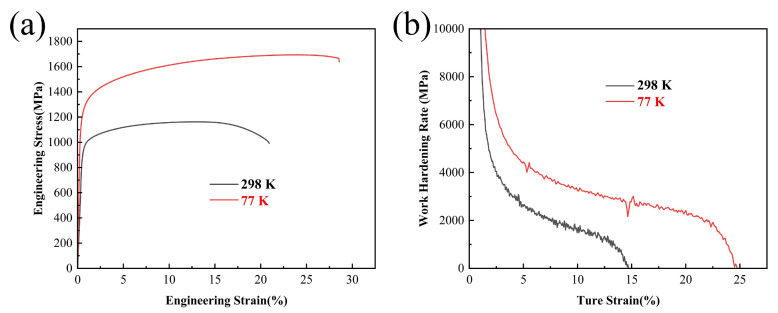
(**a**) Engineering stress–strain curves of the Al_11_Cr_14_Fe_50_Ni_25_ HEA tested at 298 K and 77 K; (**b**) strain-hardening rate as a function of strain for the Al_11_Cr_14_Fe_50_Ni_25_ HEA tested at 298 K and 77 K.

Figure 7a shows the engineering stress–strain curves of the short-time annealed Al_11_Cr_14_Fe_50_Ni_25_ high-entropy alloy tested at 298 K and 77 K. At room temperature, the alloy exhibits a favorable strength–ductility combination, with a yield strength of 953 ± 9 MPa, an ultimate tensile strength of 1160 ± 13 MPa, and an elongation to failure of 21.1 ± 1.2%. Upon decreasing the temperature to 77 K, both strength and ductility are markedly enhanced: the yield strength and ultimate tensile strength increase to 1268 ± 12 MPa and 1686 ± 15 MPa, respectively, while the elongation to failure increases to 28.6 ± 1.5%. These results demonstrate an excellent synergistic improvement in strength and ductility at cryogenic temperatures (Table 2).

Figure 7b presents the strain-hardening rate as a function of strain at 298 K and 77 K. Compared with that at room temperature, the alloy exhibits a substantially higher strain-hardening capability at 77 K and is able to maintain a relatively high strain-hardening rate over a broader strain range. This pronounced strain-hardening capacity is a key factor enabling the simultaneous enhancement of strength and ductility at low temperatures.

## 4. Discussion

To clarify the origin of the outstanding cryogenic mechanical performance of the Al_11_Cr_14_Fe_50_Ni_25_ HEA, the tensile behavior is analyzed in conjunction with the hierarchical heterogeneous microstructure revealed by SEM, EBSD, and TEM observations. The results indicate that hetero-deformation-induced (HDI) strengthening plays a dominant role in governing the deformation response, especially at 77 K.

As established in the microstructural characterization, the alloy consists of unrecrystallized and recrystallized domains, together with a metastable dual-phase architecture composed of a relatively soft FCC phase and a harder ordered B2 phase. These heterogeneous constituents differ significantly in strength, deformability, and defect density. During the early stage of plastic deformation, the FCC phase and recrystallized soft regions are expected to yield first, whereas the B2-rich domains and unrecrystallized regions, owing to their higher strength and greater resistance to plastic flow, deform less readily. This incompatibility in local deformation generates pronounced strain gradients across phase and domain boundaries, which in turn promote the accumulation of geometrically necessary dislocations (GNDs). The resulting long-range internal stress, or back stress, provides an additional strengthening contribution and enhances strain hardening, which is the characteristic feature of HDI strengthening [36,37,38].

To quantitatively evaluate the contribution of HDI strengthening, load–unload–reload (LUR) tensile tests were performed at 77 K. Following the method proposed by Yang et al. [39], the HDI stress, σ*_HDI_*, can be estimated from the unloading and reloading response according to

$$\sigma_{HDI}=(\sigma_{u}+\sigma_{r})/2$$

where *σ_u_* is the unloading stress, and σ*_r_* is the reloading yield stress.

**Figure 8:**
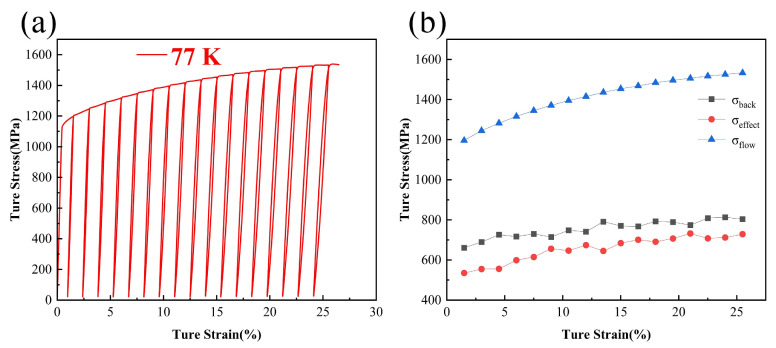
(**a**) Load–unload–reload tensile curves of the Al_11_Cr_14_Fe_50_Ni_25_ HEA tested at 77 K; (**b**) evolution of flow stress, HDI stress, and effective stress as a function of strain at 77 K.

Figure 8a,b show the representative LUR curves and the corresponding evolution of flow stress, HDI stress, and effective stress at 77 K. Both σ*_HDI_* and the effective stress (σ*_eff_*) increase continuously with increasing strain, indicating the progressive development of internal stress and dislocation resistance during deformation. Notably, at a true strain of 25%, the HDI stress reaches approximately 804 MPa, accounting for more than 52% of the total flow stress. This result clearly demonstrates that HDI strengthening is not merely a secondary contribution but rather the predominant strengthening mechanism responsible for sustaining work hardening and enabling the exceptional combination of strength and ductility at cryogenic temperatures.

The dominance of HDI strengthening can be rationalized by the hierarchical nature of the present microstructure. Unlike conventional dual-phase alloys with only phase-level heterogeneity, the Al_11_Cr_14_Fe_50_Ni_25_ HEA contains heterogeneity at multiple structural levels, including phase contrast between FCC and B2, morphological contrast between coarse and fine regions, and defect-density contrast between recrystallized and unrecrystallized domains. Such multilevel heterogeneity can intensify strain partitioning over a broad range of length scales, thereby continuously generating back stress as deformation proceeds. This mechanism is consistent with the high KAM values observed in the unrecrystallized and B2-rich regions, which indicate substantial GND accumulation associated with strain incompatibility.

Another important factor contributing to the superior cryogenic performance is the metastable microstructural state retained after short-time annealing. As discussed above, the experimentally observed phase constitution deviates significantly from the equilibrium prediction, indicating that the alloy remains in a metastable FCC-dominated dual-phase condition. This metastable state is beneficial because it combines a sufficient fraction of ductile FCC phase for plastic accommodation with hard B2 regions for strength enhancement. At the same time, the non-coherent FCC/B2 interfaces arising from the substantial lattice mismatch provide strong barriers to dislocation motion and effective sites for dislocation storage. Under cryogenic deformation, where dynamic recovery is suppressed, these interfaces can more effectively retain dislocations and thus amplify strain hardening.

**Figure 9:**
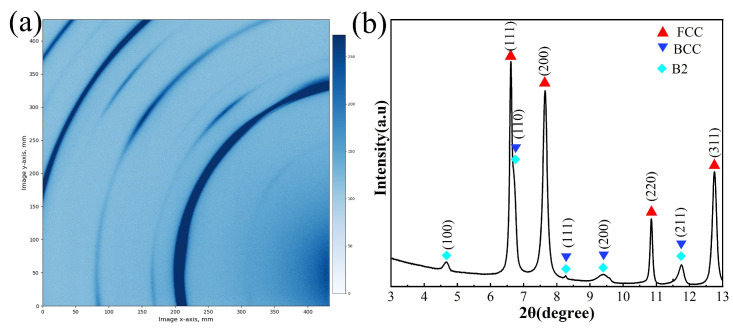
Synchrotron high-energy X-ray diffraction analysis of the phase constitution of the Al_11_Cr_14_Fe_50_Ni_25_ HEA after tensile deformation at 77 K: (**a**) two-dimensional diffraction patterns and (**b**) the corresponding integrated one-dimensional diffraction profiles.

To investigate the evolution of phase constitution during cryogenic deformation and determine whether any deformation-induced phase transformation occurred, synchrotron high-energy X-ray diffraction (XRD) was employed to characterize the Al_11_Cr_14_Fe_50_Ni_25_ HEA after tensile deformation at 77 K. Figure 9a,b present the two-dimensional diffraction patterns and the corresponding integrated one-dimensional diffraction profiles, respectively. As shown in Figure 9b, the diffraction profiles after tensile deformation at 77 K are dominated by the FCC phase, while reflections corresponding to the A2/B2 phases remain clearly visible. Quantitative phase analysis indicates that the FCC and A2/B2 phases account for approximately 71.7% and 28.3% of the total phase fraction, respectively. Notably, no new diffraction peaks emerge, nor do any existing peaks disappear after deformation, indicating the absence of significant deformation-induced phase transformation. These results demonstrate that the initial multiphase architecture remains stable throughout cryogenic tensile deformation.

A detailed comparison of the diffraction profiles before and after deformation reveals pronounced peak broadening in both the FCC and A2/B2 phases. Such peak broadening is commonly associated with the accumulation of crystal defects and lattice distortions, particularly an increase in dislocation density. Therefore, the observed broadening suggests substantial dislocation storage in both constituent phases during deformation at 77 K. The enhanced dislocation accumulation increases the resistance to subsequent dislocation motion through intensified dislocation interactions, thereby promoting pronounced strain hardening. This sustained work-hardening capability effectively delays plastic instability and contributes to the exceptional combination of strength and ductility exhibited by the alloy under cryogenic conditions.

**Figure 10:**
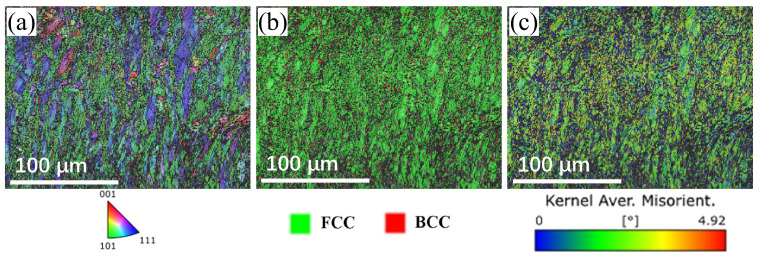
(**a**) EBSD inverse pole figure (IPF) map, (**b**) phase map, and (**c**) kernel average misorientation (KAM) map of the Al_11_Cr_14_Fe_50_Ni_25_ HEA after tensile deformation at 77 K.

Figure 10 shows the EBSD maps of the alloy after tensile deformation at 77 K. The heterogeneous microstructure, consisting of recrystallized and unrecrystallized regions, is largely retained after tensile deformation at 77 K. Meanwhile, the alloy remains predominantly composed of the FCC phase, and no evidence of significant deformation-induced phase transformation is detected. This observation suggests that the exceptionally low-temperature tensile behavior does not primarily originate from a transformation-induced plasticity mechanism but rather from the stable maintenance of heterogeneous microstructure, which promotes strain partitioning between the constituent regions and facilitates sustained geometrically necessary dislocation accumulation during deformation. The resulting hetero-deformation-induced strengthening, together with enhanced work hardening arising from dislocation storage, contributes to the exceptional combination of strength and ductility achieved under cryogenic conditions.

Although the present alloy contains FCC and A2/B2 phases with different elastic/plastic responses, the LUR-based analysis remains effective for evaluating the evolution of hetero-deformation-induced (HDI) stresses because all loading–unloading–reloading cycles were conducted under identical geometry, temperature, and strain-rate conditions, and the unloading and reloading branches exhibited highly reproducible hysteresis behavior. While the elastic-modulus mismatch between the FCC and A2/B2 phases may influence the absolute magnitude of the calculated HDI stress, it does not affect the observed evolution trend. Therefore, the calculated σ*_HDI_* should be interpreted in conjunction with the microstructural evidence rather than as a stand-alone quantitative measure.

**Figure 11:**
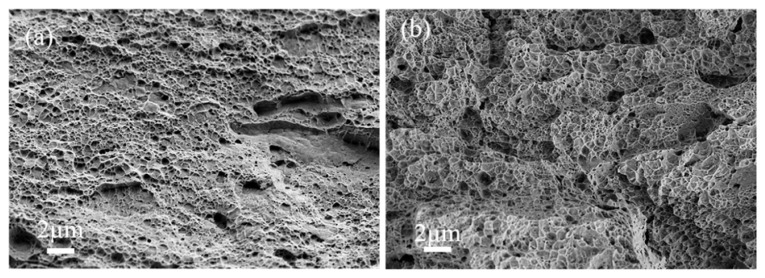
Fracture surface morphologies of the Al_11_Cr_14_Fe_50_Ni_25_ HEA after tensile testing: (**a**) 298 K; (**b**) 77 K.

Additional evidence for the enhanced plastic accommodation at low temperature is provided by the fracture morphology. Figure 11 compares the fracture surfaces of specimens tested at 298 K and 77 K. At room temperature, the fracture surface exhibits typical ductile features characterized by dimples and tearing ridges, as shown in Figure 11a. In contrast, the specimen fractured at 77 K displays a higher density of finer and more uniformly distributed dimples, as shown in Figure 11b. The refinement and homogenization of the dimple morphology indicate more uniformly distributed plastic deformation prior to fracture, which is consistent with the improved tensile ductility observed at cryogenic temperature. This fractographic evidence further supports the conclusion that the alloy undergoes more sustained and stable plastic flow at 77 K than at room temperature.

Overall, the Al_11_Cr_14_Fe_50_Ni_25_ HEA exhibits a pronounced temperature dependence in its tensile behavior. Unlike many structural alloys that suffer a strength–ductility trade-off at cryogenic temperatures, the present alloy demonstrates simultaneous improvements in both strength and ductility upon cooling from 298 K to 77 K. This exceptional cryogenic response originates from the synergistic effects of a hierarchical heterogeneous microstructure, comprising recrystallized and unrecrystallized regions, together with the dual-phase FCC + A2/B2 constitution. The heterogeneous architecture promotes strain partitioning and HDI strengthening, leading to substantial back-stress development and enhanced storage of geometrically necessary dislocations. Meanwhile, synchrotron high-energy XRD results reveal significant dislocation accumulation in both FCC and A2/B2 phases during deformation, which contributes to sustained strain hardening. The suppression of dynamic recovery at cryogenic temperatures further enhances dislocation storage and delays strain localization, thereby enabling an exceptional strength–ductility synergy. These findings demonstrate the effectiveness of hierarchical heterostructure engineering in achieving outstanding cryogenic mechanical performance in low-cost, Co-free HEAs. More broadly, this work provides valuable insights into the design of high-strength, high-ductility structural materials for cryogenic and other extreme-service environments.

## 5. Conclusions

The main conclusions of this work are summarized as follows:

(1) A Co-free Al_11_Cr_14_Fe_50_Ni_25_ high-entropy alloy with a hierarchical heterogeneous microstructure was successfully developed through Fe enrichment combined with thermomechanical processing. The resulting microstructure consists of an FCC-dominated matrix, an ordered B2 phase, and distinct recrystallized and unrecrystallized domains distributed over multiple length scales.

(2) The alloy exhibits an outstanding strength–ductility synergy, particularly at cryogenic temperatures. At 77 K, the alloy achieves a yield strength of 1268 MPa, an ultimate tensile strength of 1686 MPa, and an elongation to failure of 28.6%, all of which are markedly superior to the corresponding values at room temperature.

(3) The exceptional mechanical performance is primarily governed by HDI strengthening. Mechanical incompatibility among the heterogeneous regions and between the FCC and A2/B2 phases promotes significant strain partitioning, GND accumulation, and back-stress hardening. Quantitative LUR analysis shows that the HDI stress contributes more than 50% of the total flow stress at high strain, demonstrating its dominant role in sustaining work hardening and delaying plastic instability.

(4) The combination of a metastable FCC + A2/B2 phase constitution, non-coherent phase interfaces, and hierarchical heterogeneity provides an effective strategy for achieving superior cryogenic mechanical properties in low-cost, Co-free HEAs. The present work offers important guidance for the design of advanced structural materials for extreme low-temperature applications.

## Figures and Tables

**Table 1:** Properties of the elements.

Element	Atomic Number	Melting Point (℃)	Atomic Radius (nm)	Crystal Structure	Electronegativity	VEC
Al	13	660	0.143	FCC	1.61	3
Cr	24	1907	0.128	BCC	1.66	6
Fe	26	1538	0.126	BCC	1.83	8
Ni	28	1455	0.124	FCC	1.91	10

**Table 2:** The comparison of mechanical properties at 77 K.

Sample	Yield Strength (MPa)	Ultimate Tensile Strength (MPa)	Total Elongation (%)	Ref.
This work	1268	1686	28.6	-
Fe_49_Mn_30_Co_10_Cr_10_N_1_	1078	1630	33.5	[33]
Al_0.5_CoCr_0.8_FeNi_2.5_V_0.2_	1004	1445	17.1	[34]
Fe_48.2_Mn_30_Co_10_Cr_10_N_1.8_	1206	1620	11.0	[35]

## Data Availability

The original contributions presented in this study are included in the article. Further inquiries can be directed to the corresponding authors.
